# Real‐Time Functional Assay of Volumetric Muscle Loss Injured Mouse Masseter Muscles via Nanomembrane Electronics

**DOI:** 10.1002/advs.202101037

**Published:** 2021-07-03

**Authors:** Hojoong Kim, Young‐Tae Kwon, Carol Zhu, Fang Wu, Shinjae Kwon, Woon‐Hong Yeo, Hyojung J. Choo

**Affiliations:** ^1^ George W. Woodruff School of Mechanical Engineering College of Engineering Georgia Institute of Technology Atlanta GA 30332 USA; ^2^ Center for Human‐Centric Interfaces and Engineering Institute for Electronics and Nanotechnology Georgia Institute of Technology Atlanta GA 30332 USA; ^3^ Department for Metal Powder Korea Institute of Materials Science Changwon 51508 South Korea; ^4^ Department of Cell Biology School of Medicine Emory University Atlanta GA 30322 USA; ^5^ Wallace H. Coulter Department of Biomedical Engineering Parker H. Petit Institute for Bioengineering and Biosciences Institute for Materials Neural Engineering Center Institute for Robotics and Intelligent Machines Georgia Institute of Technology Atlanta GA 30332 USA

**Keywords:** craniofacial volumetric muscle loss (VML), electromyograms, muscle resident stem cells, nanomembrane sensors, wireless soft electronics

## Abstract

Skeletal muscle has a remarkable regeneration capacity to recover its structure and function after injury, except for the traumatic loss of critical muscle volume, called volumetric muscle loss (VML). Although many extremity VML models have been conducted, craniofacial VML has not been well‐studied due to unavailable in vivo assay tools. Here, this paper reports a wireless, noninvasive nanomembrane system that integrates skin‐wearable printed sensors and electronics for real‐time, continuous monitoring of VML on craniofacial muscles. The craniofacial VML model, using biopsy punch‐induced masseter muscle injury, shows impaired muscle regeneration. To measure the electrophysiology of small and round masseter muscles of active mice during mastication, a wearable nanomembrane system with stretchable graphene sensors that can be laminated to the skin over target muscles is utilized. The noninvasive system provides highly sensitive electromyogram detection on masseter muscles with or without VML injury. Furthermore, it is demonstrated that the wireless sensor can monitor the recovery after transplantation surgery for craniofacial VML. Overall, the presented study shows the enormous potential of the masseter muscle VML injury model and wearable assay tool for the mechanism study and the therapeutic development of craniofacial VML.

## Graphical abstract

This paper reports a wireless, noninvasive nanomembrane system that integrates skin‐wearable printed sensors and electronics for real‐time, continuous monitoring of volumetric muscle loss (VML) on craniofacial muscles. The craniofacial VML model, using biopsy punch‐induced masseter muscle injury, shows impaired muscle regeneration. Overall, the system provides highly sensitive electromyogram detection on masseter muscles with or without VML injury.

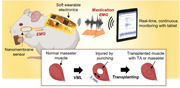

## 1 Introduction

The craniofacial region contains about 60 muscles that are vital for daily life functions, including eye movements, food uptake, respiration, and facial expressions.^[^ ^1^, ^2^ ^]^ Although head and limb muscles are comparable to contractile organs, head muscles have several unique features compared to limb muscles, including distinctive embryonic origins,^[^ ^1^, ^3^, ^4^, ^5^ ^]^ and differential susceptibility to different types of muscular dystrophies.^[^ ^6^ ^]^ Even though skeletal muscle is capable of regenerating damaged muscles via activation of muscle‐specific stem cells, called satellite cells,^[^ ^7^ ^]^ regeneration capacity varies between muscles. For example, masseter muscles that are critical for mastication have less regenerative capacity than tibialis anterior (TA) muscles^[^ ^8^ ^]^ because masseter muscles contain fewer satellite cells that show delayed differentiation compared to satellite cells of limb muscles.^[^ ^9^ ^]^ In contrast, satellite cells of other craniofacial muscles, such as extraocular muscles, show increased regenerative capacities relative to limb satellite cells.^[^ ^10^, ^11^ ^]^ Therefore, understanding the unique features of specific muscles could lead to the development of targeted therapeutic approaches for the treatment of muscle injury.

Volumetric muscle loss (VML) refers to the traumatic or surgical loss of skeletal muscle tissues, which leads to chronic muscle weakness and impaired muscle function.^[^ ^12^ ^]^ VML is often associated with military casualties as well as civilian vehicle accidents or gunshot injuries. VML is a clinically challenging problem since it requires surgical autologous muscle transplantation, which causes significant donor site morbidity.^[^ ^13^ ^]^ Therefore, many research groups have been focused on muscle regeneration using myogenic cell therapies and extracellular matrix development in an animal extremity VML model.^[^ ^14^, ^15^, ^16^, ^17^ ^]^ Among the injury‐caused VML, craniofacial injury with soft tissue penetration is a significant portion of battlefield injury^[^ ^18^ ^]^ and civilian trauma injury.^[^ ^19^ ^]^ Craniofacial VML causes loss of muscle function and severe cosmetic deformities, which may lead to social isolation and psychological depression.^[^ ^19^, ^20^ ^]^ Several works have investigated VML of sheet‐like muscles, which resemble the architecture of craniofacial muscles, using thin trunk muscles, including rat abdominal muscles^[^ ^21^, ^22^, ^23^ ^]^ and rat latissimus dorsi.^[^ ^20^, ^24^ ^]^ Studies have been conducted on VML on craniofacial muscles of large animals, such as zygomaticus muscles of sheep, emphasizing the pathophysiological differences between limb and craniofacial VML.^[^ ^25^ ^]^ However, a craniofacial VML mouse model using actual craniofacial muscles has not been reported yet due to the small size of the craniofacial muscles of a mouse. A potential challenge in developing the craniofacial VML mouse model is the lack of functional assay tools that can monitor the regeneration and recovery of injured craniofacial muscles in the active mouse in a noninvasive manner. Current existing electromyogram (EMG) systems have limitations for longitudinal study using mouse models due to the bulky system, which requires invasive metal sensors, wires, and multiple electronic components.^[^ ^26^, ^27^, ^28^ ^]^ Recent advances in wearable electronics have enabled wireless monitoring of various physiological signals that can be measured on the skin.^[^ ^29^, ^30^, ^31^ ^]^ Compact device integration on a soft elastomeric platform can provide comfortable wearability without motion artifacts caused by cumbersome wires and rigid systems.^[^ ^32^, ^33^ ^]^ The use of noninvasive and ergonomic factors in the monitoring system/device prevents restriction of movement during measurement, thus allowing us to monitor the physiological response in a natural ambulatory environment.

Here, this paper introduces nanomembrane electronics to measure real‐time muscle EMG on the skin of mouse masseter muscles with or without biopsy punch‐induced VML. We confirm that the masseter VML model shows the impaired muscle regeneration. To measure the function of VML‐injured masseter muscles in active mice, we use a wireless and wearable electronic system to provide real‐time EMG monitoring. This system includes ultrathin, low‐profile, lightweight, and stretchable membrane sensors based on biocompatible graphene and thin‐film soft circuits for data processing, which offers seamless mounting on the skin of mice without disrupting their natural behavior. In vivo demonstration of the EMG recording on the masseter muscles of mice validates the functionality of the wearable system that can clearly distinguish the signal difference between mice with and without craniofacial VML. To our knowledge, this work is the first report to use a wireless, noninvasive, soft EMG system in active and moving mice. **Table** **1** captures the novelty of our work compared to the prior reports in terms of electrode type, measurement type, recording system, target muscle, and data recording condition.^[^ ^26^, ^27^, ^34^, ^35^, ^36^, ^37^, ^38^ ^]^ In addition, this system monitors the functional recovery after transplantation surgery to treat VML. We show that there is increased fibrosis and reduced EMG activities of VML‐injured muscles following transplantation, regardless of the source of donor's muscles.

**Table 1:** Comparison of rodent model study with EMG measurements

Reference	Electrode type	Noninvasive electrode	Data transmission	Target muscle	Recording condition	Application
This work	Stretchable graphene membrane	Yes	Wireless	Masseter muscles	Freely moving and mastication	Monitoring of craniofacial VML and functional recovery with transplanted muscles
Kompotis et al.^[^ ^27^ ^]^	Flexible gold wires	No	Wire	Trapezius muscles	During sleep	Identifying the effect of rocking on sleep quality
Burns et al.^[^ ^36^ ^]^	Rigid stainless‐steel needle	No	Wire	Diaphragm and external intercostal muscles	Anesthetized	Evaluating diaphragm dysfunction by respiratory muscle weakness
Silvani et al.^[^ ^37^ ^]^	Flexible stainless‐steel wire	No	Wire	Neck and tibialis anterior muscles	During sleep	Monitoring of muscle activity during sleep
Ahmed^[^ ^35^ ^]^	Rigid stainless‐steel needle	No	Wire	External urinary sphincter muscles	Anesthetized	Monitoring of lower urinary tract with spinal cord injury
Freedman et al.^[^ ^26^ ^]^	Rigid stainless‐steel needle	No	Wire	Facial muscle	Freely moving	Monitoring of optogenetic behavior of mice
Hadzipasic et al.^[^ ^38^ ^]^	Rigid borosilicate glass	No	Wire	lower leg flexor and extensor muscles	Limited moving	Identifying motor neuron loss of mice with amyotrophic lateral sclerosis
Sicari et al.^[^^34]^	Flexible stainless‐steel wire	No	Wire	Tensor fascia latae muscles	Anesthetized	Evaluating the healing of VML by extracellular matrix scaffold

## 2 Results and Discussion

### 2.1 Overview of Real‐Time Functional Assay of Craniofacial VML in Mouse Using Wireless Nanomembrane Electronics

**Figure 1:**
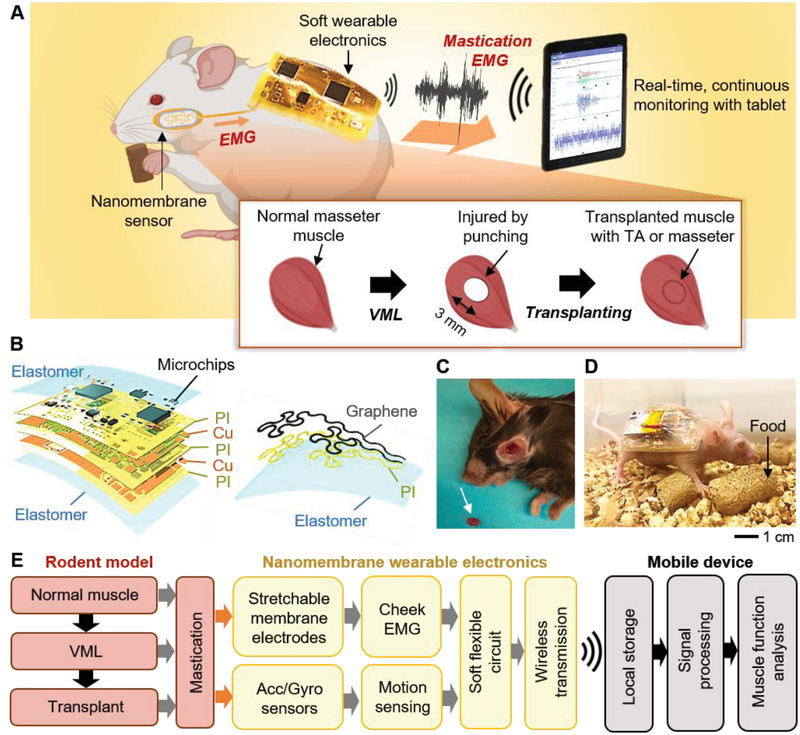
Overview of real‐time functional assay of craniofacial VML in mouse using wireless nanomembrane electronics. A) Schematic illustration of a wireless electronic system on the skin of a mouse for a functional quantification of craniofacial VML of the masseter muscle. The mouse's EMG of the cheek area is continuously monitored on the mobile device. Inset shows the process of punch‐induced VML of masseter and transplantation to treat VML of the masseter. Masseter muscle was injured by biopsy punching as a model of craniofacial VML. VML‐injured masseter muscles are transplanted with a biopsied piece of tibialis anterior (TA) or masseter muscles to fill the injured area. B) Schematic illustration of a multilayered structure of a soft circuit (left) and stretchable sensor (right). C) Muscle biopsy to create VML in masseter muscles. The middle of the masseter muscles is biopsied by a 3 mm biopsy punch. Arrow indicates a muscle piece from a biopsy. D) Optical image of an active mouse with the device during mastication. E) Flow chart capturing the quantitative metrics of analyzing muscle function.

To monitor the change of muscle activities during mastication in active mice, we developed a wireless, nanomembrane electronic system (**Figure** **1**A). This device includes noninvasive, stretchable electrodes to apply directly on the cheek skin of a mouse and a miniaturized, wireless, soft circuit to attach to the back of a mouse. During mastication, EMG data are measured by the electrodes on the mouse cheek skin and delivered to the microprocessor in the circuit for data processing. The signals are then wirelessly transmitted to an external mobile device for real‐time, continuous data monitoring and saving. To establish VML on masseter muscles, a normal masseter muscle is injured by a 3 mm diameter biopsy punch. The injured muscle area is transplanted with 3 mm biopsied TA or masseter muscle (inset of Figure 1A). Figure 1B presents a schematic illustration of a multilayered soft circuit architecture (left image) and serpentine‐patterned electrodes (right image) on a soft elastomeric substrate, allowing for a conformal lamination on the mouse skin. Figure 1C displays the muscle biopsy used to produce masseter VML, while Figure 1D shows that movement remains unhindered during monitoring. The flow chart shown in Figure 1E denotes the quantitative metrics for wireless EMG measurement and analyzing muscle function. This wearable electronic system can monitor EMG activities in each of the three different craniofacial muscle conditions (normal, VML, treated VML by transplant) in our rodent model. Also, the motion sensor package using a sensitive accelerometer and gyroscope, which identifies the mouse movements to distinguish mastication motions. A mobile device embeds a custom‐designed app to offer real‐time signal displaying and data storing to analyze muscle functions.

### 2.2 Characterization of Graphene Membrane Electrodes and Soft Wireless Circuits

**Figure 2:**
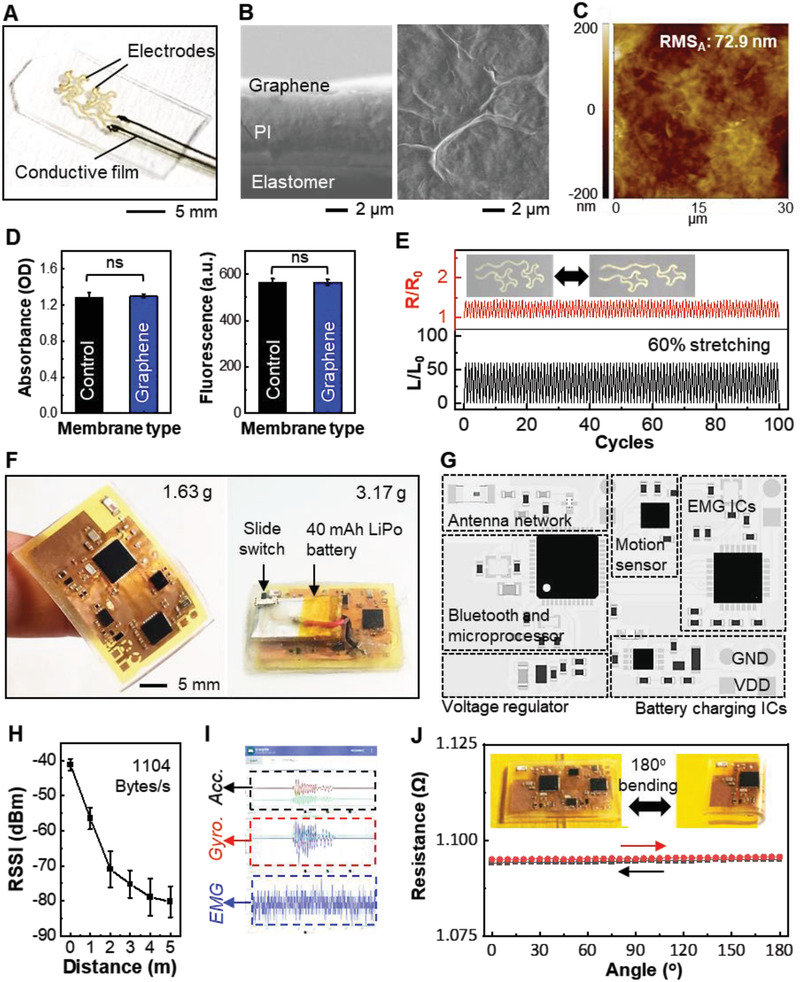
Characterization of graphene membrane electrodes and wireless soft circuits. A) Photo of stretchable printed electrodes on an elastomeric membrane. B) Cross‐sectional (left) and top‐view (right) SEM image of the multilayered electrodes. C) AFM image indicating the surface roughness of the printed graphene. The average RMS is 72.9 nm, which captures the uniformity in the printed graphene layer. D) Comparison of cell absorbance (left) and fluorescence (right) of cultured cells on the control and graphene. Data is analyzed 1‐way analysis of variance (ANOVA) (ns = not statistically significant). Error bars represent standard error. E) Graph representing relative resistance variation (top) according to the 60% of tensile strain change (bottom) for 100 cycles. F) Optical image that captures a miniaturized, lightweight, soft circuit (left). The circuit's total weight is 3.17 g, including a rechargeable battery (40 mAh capacity) with a slide switch (right image). G) Illustration of the circuit design with multiple functional electronic components. H) RSSI response according to the Bluetooth communicating distance, showing maintained data transmission rate 1104 bytes s^−1^. I) Mobile device application interface that displays real‐time, continuous motion (accelerometer and gyroscope) and EMG signals from a wearable sensor system. J) Resistance change of the flexible circuit upon cyclic loading (100 times with 180° bending at 1.5 mm radius of curvature), showing a negligible change of resistance.

To quantify the muscle function of our craniofacial VML model, we monitored EMG signals with a wearable wireless system. Conventional EMG systems in animal studies^[^ ^35^, ^38^, ^39^ ^]^ use bulky, invasive, needle‐type‐wired electrodes that penetrate into target muscle. The main issue of this system is that it is unsuitable for use in small mice with active movements. This study developed a noninvasive, miniaturized, soft electronic system to offer a wireless, high‐fidelity recording of muscle functions with naturally moving mice. **Figure** **2**A shows an example of printed stretchable electrodes on a soft elastomeric substrate. We utilized a nanomanufacturing process to fabricate the skin‐wearable electrodes. Two electrodes are manufactured by aerosol jet printing (AJP) in a serpentine shape to maximize stretchability. Conductive flexible films make connections between the sensor and the soft circuit. The width of the printed graphene membranes was 0.55 mm. Printed graphene and polyimide (PI) membranes are well‐stacked on the elastomer (left image in Figure 2B), and graphene sheets form a complete film without boundaries for enhanced conductivity (right image in Figure 2B). With the low‐profile graphene membrane and PI layer inserted under the graphene, the electrodes can endure mechanical deformation during the fabrication and measurement process. We confirmed that there are no film damages or cracks on the graphene film. The image from atomic force microscopy (AFM) captures the printed graphene layer's uniform morphology (Figure 2C). The details of the printing process are shown in Figure S1 (Supporting Information). The biocompatibility of a tissue‐mounted sensor is a critical feature to guarantee safe and continuous use with adverse effects.^[^ ^40^, ^41^ ^]^ In addition, cytotoxicity of the electrode can damage the skin cells of the VML‐injured region when measuring muscle activities. Biocompatible characterizations for the printed graphene electrodes were conducted with human keratinocyte cells. The number of live cells on the graphene and control (polystyrene cell culture dish) was determined via fluorescence intensity (Figure S2, Supporting Information). The absorbance (left graph) and fluorescence (right graph) of cultured cells in Figure 2D reveal that the printed graphene electrodes have a negligible influence on cell viability. Figure 2E represents the relative resistance variation of the electrodes (top) according to the 60% of tensile strain change (bottom) for 100 cycles, showing a reliable mechanical performance under strain. Figure 2F shows optical images that capture miniaturized, lightweight structures of a soft circuit (left image). The circuit has a small dimension (6 cm^2^) and thickness (< 2 mm) and is extremely lightweight (1.63 g). After integrating with a rechargeable battery (40 mAh capacity) with a slide switch, the total weight of the circuit becomes 3.17 g. The small battery allows the active wireless system to record multiple signals over 6 h continuously (Figure S3, Supporting Information). Figure 2G shows the circuit design with integrated functional components, including a Bluetooth microprocessor that can deliver the measured EMG and motion signals to a mobile device. A detailed description of the components is shown in Figure S4 (Supporting Information). Analysis of received signal strength indication (RSSI) shows a successful wireless communicating distance of 5 m with a maintained data transmission rate of 1104 bytes s^−1^ (Figure 2H). The transmitted EMG and motion signals are displayed and stored in a mobile device with a customized app (Figure 2I). The soft, flexible circuit demonstrates mechanical reliability even under complete folding (180° with 1.5 mm radius of curvature) during the cyclic loading (100 cycles; Figure 2J), which agrees with the results from the computational modeling data (Figure S5, Supporting Information).

### 2.3 Establishment of Craniofacial VML and Defective Masseter Muscle Regeneration

**Figure 3:**
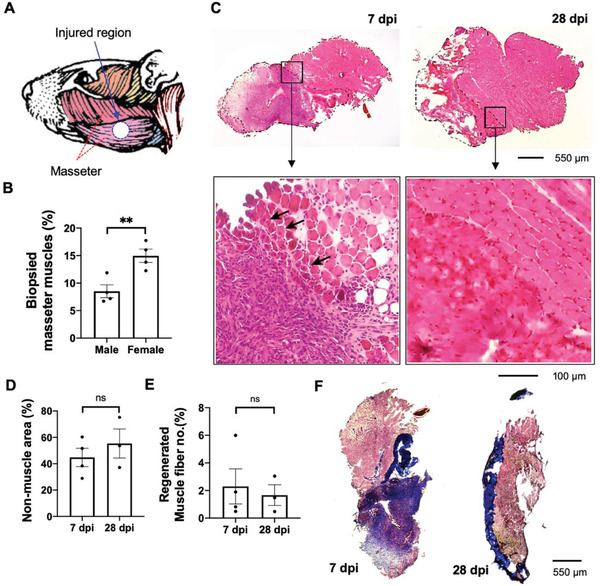
Establishment of craniofacial VML and defective masseter muscle regeneration. A) Illustration of craniofacial VML of masseter muscle of a mouse. B) 3 mm biopsy punches induce different degree of muscle loss between 6‐month‐old male and females. *n* = 4 for each sex. C) Histology of masseter muscle after 7 days (left) and 28 days (right) post VML injury (dpi). Muscle sections were stained with Hematoxylin and Eosin to visualize nucleus (purple) and cytosol (red). Dotted area indicates nonmuscle area. Small squared images are enlarged at the lower panels to show cellular elements of muscle tissues. Arrows in lower panel indicate muscle fiber containing central nucleus, which is a feature of regenerating muscle fiber. D) Non‐muscle area occupies majority of masseter muscles at 7 and 28 days postinjury. Error bars represent standard error of the mean (SEM). E) Number of regenerated muscle fibers is very low at 7 and 28 days postinjury. Data are analyzed by Student *t*‐test. ns = not significant statistically. F) Fibrosis of masseter muscle after 7 (left) and 28 days (right) post VML injury. Muscle sections were stained with Massion's Trichrome staining to visualize fibrotic area (blue) from muscle tissues (brown).

To establish craniofacial VML, we chose masseter muscles that are key muscles for mastication by pulling the mandible upward. Masseter muscles are composed of superficial and deep masseter muscles. The superficial masseter muscle is the thick and tendon‐like portion and connects to the cheekbone, while the deep masseter muscle is smaller and connects to the mandible.^[^ ^42^ ^]^ We injured superficial masseter muscles using a 3 mm biopsy punch (**Figure** **3**A), which can generate about 8.5% and 15% loss of masseter muscle tissues by losing 8.5 ± 2.3 and 7.9 ± 1.2 g of muscle masses from 6 months‐old male and female mice, respectively (Figure 3B). Masseter VML injury using a 3 mm biopsy on female mice presented a similar critical injury size (loss of 15%) volume compared to mouse limb VML injury,^[^ ^16^ ^]^ which was not the case for male mice, although 3 mm biopsy on masseter muscles of male mice would be possible to induce functional impairment, we decided to use female mice for the remainder of the experiments. To validate whether muscle regeneration occurs post‐3 mm muscle biopsy induced injury in a craniofacial VML model, we sectioned masseter muscles at 7 and 28 days of the post‐VML injury. Figure 3C shows histology data of a midpoint of the VML injury of masseter muscles colored through Hematoxylin and Eosin staining, which shows that nonmuscle areas (dotted lines in Figure 3C) are filled with nonmuscle cells, maybe immune cells, and fibrosis. The nonmuscle area occupied around 40% of injured masseter muscles at 7 days postinjury (dpi) and remained at 28 dpi (Figure 3D). We also observed very limited muscle regeneration at the rim of injured areas by measuring the cross‐sectioned area of muscle fibers containing central nuclei, a feature of regenerated muscles (arrows in the bottom image of Figure 3C). The regenerated muscle fiber number was about 2% of total muscle fibers in masseter muscles in 7 and 28 dpi (Figure 3E). Additionally, fibrosis in nonmuscle areas of masseter muscles postday 7 and day 28 VML injury was detected by Massion's Trichrome staining (Figure 3F). Defective muscle regeneration after VML was supported by reduced satellite cells of masseter muscles with VML (Figure S6, Supporting Information), which are essential muscle stem cells for muscle regeneration upon injury^[^ ^7^ ^]^ compared to ones of freeze‐induced masseter muscle injury. In addition, fibroadipose progenitor cells, a muscle mesenchymal stem cell which mediates fibrosis and fat deposit in chronic muscle injury,^[^ ^43^, ^44^ ^]^ were highly increased in VML masseter muscles than freeze‐induced masseter muscle injury (Figure S7, Supporting Information), which may induce fibrosis in VML masseter muscles. These results prove that craniofacial VML generated by a 3 mm biopsy injury results in defective muscle regeneration similar to what is seen in limb VML experiments using the same method.^[^ ^16^ ^]^

### 2.4 Demonstration of a Wireless, Wearable EMG System to Assess the Functionality of VML‐Injured Masseter Muscles

**Figure 4:**
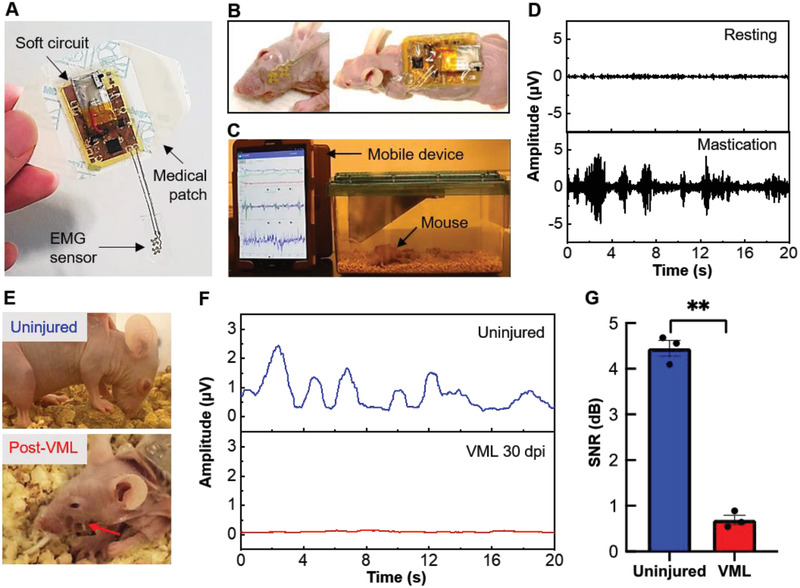
Wireless, wearable EMG system to assess the functionality of VML‐injured masseter muscles. A) Fully integrated wireless wearable electronics on a thin‐film medical patch. B) Photos showing an ultrathin stretchable EMG sensor attached on the cheek (left) and a soft circuit on the back (right) of a nude mouse. C) Photo of the experimental setup with a mouse in a cage. Real‐time, continuous motion, and EMG data are monitored and recorded by a mobile device with an embedded application. Movie S1 (Supporting Information) shows an example of wireless, real‐time EMG monitoring with wearable electronics. D) Comparison of real‐time EMG signals, measured with an uninjured mouse, during resting state (top) and mastication phase (bottom), which shows clear signal differences. E) Photos of an uninjured mouse (top) and post‐VML‐injured mouse after 30 days (bottom). Arrow indicates the location of the VML‐injured area. F) Representative RMS EMG signals during mastication corresponding to two cases in E). A clear signal difference is observed between the uninjured case (top) and VML‐injured masseter muscles (bottom) at 30 days postinjury. G) Summarized EMG SNR data between the uninjured and post‐VML‐injured masseter muscles during mastication. Data were analyzed by the unpaired two‐tailed student *t*‐test. **p < 0.01.

**Figure****4**A represents an integrated, ultrathin wearable electronic system for the noninvasive diagnosis of VML in mice. The device is fully covered with a thin‐film patch to easily mount on the skin of a target mouse (Figure 4B). Stretchable EMG electrodes are directly attached to the mouse's cheek (left image in Figure 4B), while the soft wireless circuit is mounted on the back of the body (right image in Figure 4B). During the mouse's natural activities, including eating and roaming in the cage, real‐time, continuous muscle functions are monitored by the wearable device and tablet (Figure 4C). Figure 4D captures real‐time EMG data, recorded from a normal mouse during the resting state (top) and mastication (bottom), which displays an increased peak‐to‐peak voltage. The amplitude of mastication EMG signals is much smaller compared to typical intermittent motion artifacts measured by the mouse (Figure S8, Supporting Information). To quantify the function of post‐VML‐injured muscle, we measured the EMG activity of masseter muscle at 30 days post‐VML injury (Figure 4E). Results show that during both resting and mastication, post‐VML mice have smaller EMG signals than uninjured mice (Figure S8, Supporting Information). In addition, both root‐mean‐squared (RMS) EMG signals (Figure 4F) and their signal‐to‐noise ratio (SNR) values (Figure 4G) indicate a large significance (*p*‐value < 0.01) between the two groups, suggesting that the VML‐injured muscle has not recovered a month after injury, which is consistent with our histology study results in Figure 3.

### 2.5 Functional Recovery Monitoring of Post‐Transplantation of VML‐Injured Masseter Muscles

**Figure 5:**
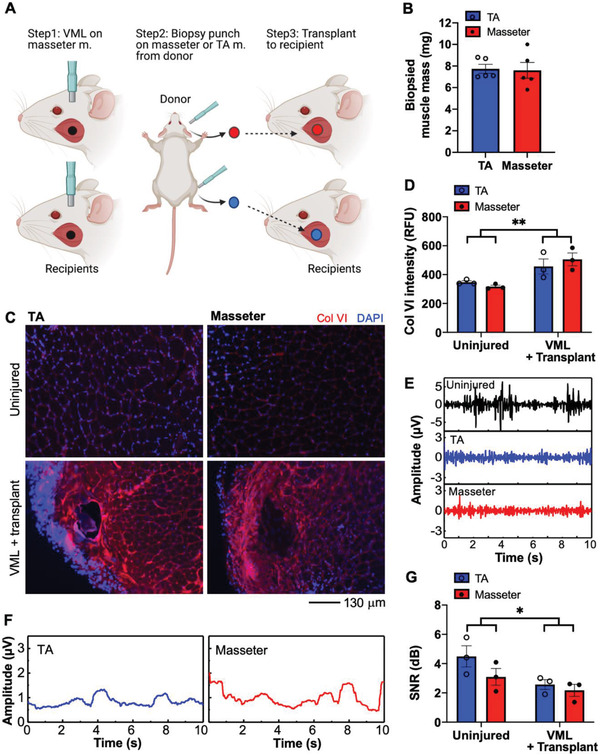
Monitoring of the functional recovery of VML‐injured masseter muscles post‐transplantation. A‐B) Experimental scheme showing biopsy‐punch induced VML area (A) of masseter muscles of immuno‐deficient mice (NRG) filled with a biopsied piece from TA (blue) or masseter (red) muscles of wild‐type mice (B). EMG measurement and fibrosis analysis are conducted at 30 days of post‐VML injury/transplantation. C) Uninjured and VML‐injured masseter muscles with TA and masseter muscle transplantation are sectioned and labeled with Col VI antibodies (red) to measure fibrosis. DAPI staining is used to label nuclei of muscle sections. D) The averaged intensity of Col VI staining indicates that transplantation of TA or masseter muscle produced a comparable level of fibrosis in transplanted VML‐injured masseter muscle. *n* = 3 for each group, and error bars represent the standard error of the mean. Data are analyzed with 2‐way ANOVA. p** < 0.01. E) Comparison of filtered EMG of uninjured (top) and the VML‐injured masseter muscles with TA (middle) or masseter (bottom) muscles transplant. F) RMS‐EMG signals measured from TA (left) and masseter (right) muscle‐transplanted masseter muscles. G) SNR values are decreased in VML with transplantation muscle group compared with uninjured muscles (*n* = 3). TA or masseter muscle transplantation produces similar levels of functional recovery of VML‐injured masseter muscles. Data are analyzed with 2‐way ANOVA. p* < 0.05.

Craniofacial VML has been treated with autologous limb muscle transplantation in the clinic.^[^ ^45^, ^46^, ^47^ ^]^ To evaluate the origins of transplanted muscles for craniofacial VML treatment, we performed transplantation of masseter or TA muscles to the VML area of masseter muscles (**Figure** **5**A). We biopsied masseter or TA muscles from wild‐type mice using 3 mm biopsy punch and transplanted them into VML (3 mm biopsied area to ensure 1:1 volume match (Figure 5B; and Figure S9, Supporting Information)) of the masseter of severely immune‐deficient mice. These mice have been used in this transplant study due to the minimal likelihood of transplant rejection from the host's immune system.^[^ ^48^ ^]^ Whole biopsied masseter or TA muscles were placed to align fibers between donors and recipients muscles to ensure proper muscle contractility. EMG activity of the transplanted masseter muscles was measured 30 days after surgery. To measure fibrosis, muscle sections from uninjured and VML‐injured masseter muscles with TA and masseter muscle transplantation were labeled with anti‐collagen VI (Col VI) antibodies (red) (Figure 5C). The intensity of Col VI signals was measured to estimate the degree of fibrosis in uninjured (contralateral) and VML‐injured masseter muscle with transplantation (Figure 5D). Fibrosis level is significantly higher in VML with transplanted masseter muscles compared to uninjured contralateral masseter muscles. However, transplantation of TA or masseter muscle does not produce a different level of fibrosis in transplanted VML‐injured masseter muscle. Also, EMG activity is monitored with wearable membrane electronics to identify the functional recovery of masseter muscles after different muscle transplantation (Figure S9, Supporting Information). Decreased EMG responses were seen in both transplanted muscles compared to contralateral muscles during mastication (Figure 5E). The signal amplitude of both transplanted muscles appears comparable (Figure 5F). The summarized SNR values in Figure 5G show that muscle function in both VML/transplanted masseter muscles is partially recovered compared to the uninjured contralateral masseter muscles. Taken together, TA or masseter muscle transplantation produces similar levels of fibrosis and functional recovery of VML‐injured masseter muscles.

## 3 Discussion

Although the current study achieved meaningful continuous EMG monitoring in mice, motion artifacts affected signal analysis. Despite the low‐profile and soft membrane electrodes, the device's size was slightly bigger than the target muscle. Also, we observed that a testing mouse occasionally attempted to scratch the device, which can be resolved by further miniaturizing a circuit and sensor for the 2nd‐generation device.^[^ ^49^, ^50^ ^]^ Additionally, we introduced a motion sensor to exclude EMG signals at high motion activity to collect the EMG activity during mastication. An algorithm based on machine learning could offer automated signal discrimination and behavioral classification for further study.^[^ ^51^, ^52^ ^]^ Since the EMG signal could be slightly different depending on the area to which the electrode was attached, constant localization with increasing the number of samples was demanded accurate results. Nevertheless, the newly developed wireless EMG system demonstrated enough sensitivity to determine the function of masseter muscles. We performed transplant experiments to verify the effectiveness of the craniofacial reconstitution surgery with autologous limb muscle graft^[^ ^45^, ^46^, ^47^ ^]^ using EMG sensors. Although we expected to obtain better outcomes (such as higher EMG), if VML of masseter muscles were transplanted with masseter muscles due to recovery of original muscle type (type I for masseter) as well as resident stem cells, veins, and nerves, the results are comparable when VML of masseter muscles were transplanted with limb muscles (TA, majority of muscle is type IIa/b). However, the fibrotic tissues around transplanted muscle tissues or cellulose tissues of the skin incision area would be huddles of accurate EMG measurements. This result again emphasized that precise sensor localization and increasing the number of samples are necessary for more accurate results. Overall, the device produced statistically different signals to distinguish between normal, the VML injured, and transplanted VML injured masseter muscles.

## 4 Conclusion

Collectively, the presented work introduces the development of a small rodent craniofacial VML model using biopsy punch and validated muscle functions with a noninvasive wearable sensor system. To detect the function of masseter muscles with active mice, we developed a fully portable, wireless electronic system that allowed for continuous EMG activity monitoring in mouse muscles, which is a widely used vertebrate animal model in muscle biology. Ultrathin, low‐profile, biocompatible nanomaterials integrated with a soft, lightweight platform mounted on the skin of mice allowed for seamless muscle function analysis without behavioral restriction. Furthermore, the miniaturized wireless system proved its potential for noninvasive and continuous diagnosis of surgical treatment in a microscale domain. To evaluate the device with a mouse model, we demonstrated that mice with craniofacial VML from a 3 mm biopsy injury in their masseter muscle displayed completely defective muscle function as well as muscle regeneration. We successfully validated the ability of this EMG monitoring system to detect defective muscle function of VML‐injured muscles as well as functional recovery of transplanted VML‐injured muscles of free‐moving mice.

## 5 Experimental Section

### Mice Used in the Study

C57BL/6J mice (Jax000664) (female *n* = 6 and male *n* = 4), *Pax7* ^CreERT2/CreERT2^ mice (Jax017763), *Rosa* ^tdTomato/tdTomato^ (tdTomato) (Jax007909), NU/J (Jax002019) (female *n* = 7), NRG (NOD.Cg‐Rag1^tm1Mom^Il2rg^tm1Wjl^/SzJ; Jax007799) (female *n* = 6) were purchased from Jackson Laboratories (Bar Harbor, ME; www.jax.org). Five to six months old mice were used, as noted in the figure legend. Homozygous *Pax7* ^CreERT2/CreERT2^ male mice were crossed with homozygous *Rosa* ^tdTomato/tdTomato^ (tdTomato) to obtain *Pax7* ^CreERT2^ *^/+^*; *Rosa* ^tdTomato/+^ (*Pax7 Cre* ^ERT2^‐*tdTomato*) mice (female *n* = 12). To label satellite cells with red fluorescence (tdTomato), tamoxifen, 1 mg (Sigma‐Aldrich, St. Louis, MO) per 10 g body weight, was injected intraperitoneally once daily for 5 days. Experiments were performed in accordance with approved guidelines and ethical approval from Emory University's Institutional Animal Care and Use Committee and in compliance with the National Institutes of Health.

### Muscle Tissue Injury and Preparation for Histology Analysis

Mice were anesthetized by 2.5% isoflurane inhalation using a nose cone. For analgesia, mice were injected subcutaneously with 0.1 mg kg^−1^ buprenorphine SR (sustained release for 3 days) before muscle injury. The target injury area is an upper part of the superficial masseter muscle. For VML injury, masseter muscle was punched by 3 mm muscle biopsy punch, which was used to generate a critical size of VML in quadriceps muscles of mouse,^[^ ^16^ ^]^ by pushing biopsy punch down until it touched with a mandible bone. To avoid bleeding, the injury area was selected to avoid cutting the external carotid artery or posterior facial vein, both of which surround the upper and lower parts of deep and superficial masseter muscles, respectively. For freeze injury, a dry ice‐cooled 4 mm metal probe was placed on the masseter muscles for 5 s as described previously.^[^ ^8^ ^]^ For transplantation surgery, the masseter muscles of NRG mice (recipient) were punched by a 3 mm muscle biopsy punch. Then, the biopsied area was filled with a biopsied piece of TA or masseter muscles from C57BL/6 mice (donor). Mass of biopsied pieces of TA and master muscles were equivalent. After injury or surgery, the skin was closed using absorbable suture. Animals were euthanized by an overdose of isoflurane at the indicated time points. Superficial masseter muscle tissues were dissected and frozen in Tissue Freezing Medium (Triangle Biomedical Sciences) and stored at −80 °C. Tissue cross‐sections of 10 µm thickness were collected every 200 µm using a Leica CM1850 cryostat. To observe muscle histology, muscle sections were stained with hematoxylin and eosin (H&E) following the manufacture's instruction, imaged with Echo Revolve widefield microscope and analyzed using ImageJ. To detect fibrosis of muscle section, slides were stained using Massion's Trichrome Staining kit (Thermo Scientific) following the manufacture's instruction (Advanced Microwave Staining Protocol). Slides were rehydrated in PBS before staining and imaged using Echo Revolve widefield microscope. To measure fibrosis in muscle tissues, muscle sections were immunostained with anti‐collagen VI antibodies (Fitzgerald Industries International, 70R‐CR009X, 1:300) and visualized AF594‐conjugated donkey anti‐rabbit antibodies. 4′,6‐diamidino‐2‐phenylindole (DAPI) was used for nuclear staining.

### Flow Cytometry for Cell Analysis

To analyze the number of satellite cells and fibroadipose progenitor cells (FAPs) in injured muscles, muscles were dissected and digested with dispase II and collagenase II as previously described.^[^ ^53^ ^]^ Isolated mononucleated cells were immunostained with the following antibodies: 1:400 CD45‐PE (clone 30‐F11; BD Biosciences), 1:4000 Sca‐1‐PE‐Cy7 (clone D7, BD Biosciences), 1:400 CD31‐PE (clone 390; eBiosciences). Fibroadipose progenitor cells were counted using the following criteria: CD31^−^/CD45^−^/Sca1^+^ and satellite cells are counted by tdTomato^+^ using BD LSR II cytometry analyzer and analyzed using FCS Expression 6 Flow software 6.01.

### Fabrication of a Nanomembrane Electronic System

The integration of a soft platform with a microfabrication technique enabled all‐in‐one, wireless, and portable electronics. The device fabrication utilized multiple nanomanufacturing techniques, including a high‐resolution printing process for graphene membrane electrodes^[^ ^52^, ^54^ ^]^ and conventional photolithography, a metallization process for a thin‐film‐based circuit.^[^ ^55^, ^56^ ^]^ Detailed description for the entire fabrication process was introduced in Note S1 and Figure S10 (Supporting Information). For electrode fabrication, PI and graphene membranes were sequentially printed as a serpentine‐patterned shape via AJP (Aerosol Jet 200, Optomec) on the polymethyl methacrylate (PMMA)‐coated glass slide. For the circuit construction, PI─Cu─PI─Cu─PI multilayers were stacked on a polydimethylsiloxane (PDMS)‐coated 4 in. wafer. The fabricated circuit and electrodes were retrieved from the carrier substrates and transferred to a soft silicone elastomer (1:1 mixture of Ecoflex 00‐30 and Gels, Smooth‐On). Functional microchips were soldered on the exposed Cu pads on the circuit and covered with the elastomer. A rechargeable LiPo battery (40 mAh, Adafruit) was integrated to the circuit. The electrodes and the circuit were linked with a flexible conductive film. A medical film (Tegaderm, 3M) was utilized to cover the device, which not only helped fix it onto a mouse's skin but also prevented external damages.

### Mouse Preparation to Use Wearable Electronics

To acclimate mice to wearing the device, mice carried dummy circuits for 2–4 h on their back a day before the experiment. To stimulate food consumption, food and water were removed for 18 h before the experiment. On experiment day, mice were anesthetized with 2.5% isoflurane inhalation using a nose cone. If necessary, hair from the cheek and back area was removed with hair‐removing lotion and wiped with alcohol pads to ensure alignment of membrane sensor or device with skin. Mice recovered on a heating pad after wearing the sensor and device. Three food pellets were provided when mice are active. The eating activity was recorded as a reference to EMG signals.

### Signal Processing and Quantifying EMG Signals

Masseter muscle EMG activity during mastication was selected for analysis. The EMG activity with a high motion signal was excluded from motion artifacts. Raw EMG signal was filtered by second‐order Butterworth bandpass filter at a cutoff frequency from 10 to 30 Hz. The filtered EMG was converted to the RMS signal to determine the peak amplitude and noise. The SNR was calculated as follows^[^ ^52^, ^57^ ^]^

(1) $$\mathrm{SN}R_{dB}=\;10\log_{10}\left[{\left(\frac{A_{\mathrm{signal}}}{A_{\mathrm{noise}}}\right)}^{2}\right]$$

where *A*_signal_ is the amplitude of RMS EMG at chewing and *A*_noise_ is the amplitude during noneating. The SNR was collected at 5 times and averaged for analysis.

### Statistical Analyses

Statistical analysis was performed using Prism 8.0. Results are expressed as the means ± SEM. Experiments were repeated at least three times unless a different number of repeats is stated in the legend. Statistical testing was performed using the unpaired *t*‐test (Welch's *t*‐test) if two groups were compared, 1‐way ANOVA analysis and Kruskal–Wallis method for posthoc comparison, if more than two groups were compared, or 2‐way ANOVA analysis if samples with 2 independent variables were compared, as stated in the figure legends. *p* < 0.05 was considered statistically significant. The statistical method, *p*‐values, and sample numbers are indicated in the figure legends. Power analysis of animal experiments was performed (Table S1, Supporting Information).

## Conflict Of Interest

W.‐H.Y. and H.J.C. are the inventors of a pending US patent application.

## Supporting information

Supporting Information

Supplemental Movie 1

## Data Availability

Data of this study are available from the corresponding author on request.

## References

[1] D. M. Noden, P. Francis‐West, Dev. Dyn. 2006, 235, 1194. 16502415 10.1002/dvdy.20697

[2] F. Wachtler, M. Jacob, Bibl. Anat. 1986, 29, 24. 3729921

[3] P. Bailey, T. Holowacz, A. B. Lassar, Curr. Opin. Cell Biol. 2001, 13, 679. 11698183 10.1016/s0955-0674(00)00271-4

[4] R. C. Mootoosamy, S. Dietrich, Development 2002, 129, 573. 11830559 10.1242/dev.129.3.573

[5] B. Christ, C. P. Ordahl, Anat. Embryol. 1995, 191, 381. 10.1007/BF00304424 7625610

[6] A. E. Emery, BMJ 1998, 317, 991. 9765171 10.1136/bmj.317.7164.991 PMC1114045

[7] C. Lepper, T. A. Partridge, C. M. Fan, Development 2011, 138, 3639. 21828092 10.1242/dev.067595 PMC3152922

[8] G. K. Pavlath, D. Thaloor, T. A. Rando, M. Cheong, A. W. English, B. Zheng, Dev. Dyn. 1998, 212, 495. 9707323 10.1002/(SICI)1097-0177(199808)212:4<495::AID-AJA3>3.0.CO;2-C

[9] Y. Ono, L. Boldrin, P. Knopp, J. E. Morgan, P. S. Zammit, Dev. Biol. 2010, 337, 29. 19835858 10.1016/j.ydbio.2009.10.005 PMC2806517

[10] L. K. McLoon, K. M. Thorstenson, A. Solomon, M. P. Lewis, Oral. Dis. 2007, 13, 134. 17305613 10.1111/j.1601-0825.2006.01353.x

[11] P. Stuelsatz, A. Shearer, Y. Li, L. A. Muir, N. Ieronimakis, Q. W. Shen, I. Kirillova, Z. Yablonka‐Reuveni, Dev. Biol. 2015, 397, 31. 25236433 10.1016/j.ydbio.2014.08.035 PMC4309674

[12] B. F. Grogan, J. R. Hsu, J. Am. Acad. Orthop. Surg. 2011, 19, S35. 21304045 10.5435/00124635-201102001-00007

[13] C. H. Lin, Y. T. Lin, J. T. Yeh, C. T. Chen, Plast. Reconstr. Surg. 2007, 119, 2118. 17519710 10.1097/01.prs.0000260595.85557.41

[14] X. Wu, B. T. Corona, X. Chen, T. J. Walters, BioRes. Open Access 2012, 1, 280. 23515319 10.1089/biores.2012.0271 PMC3559228

[15] K. Garg, C. L. Ward, B. J. Hurtgen, J. M. Wilken, D. J. Stinner, J. C. Wenke, J. G. Owens, B. T. Corona, J. Orthop. Res. 2015, 33, 40. 25231205 10.1002/jor.22730

[16] S. E. Anderson, W. M. Han, V. Srinivasa, M. Mohiuddin, M. A. Ruehle, J. Y. Moon, E. Shin, C. L. San Emeterio, M. E. Ogle, E. A. Botchwey, N. J. Willett, Y. C. Jang, Tissue Eng., Part C 2019, 25, 59. 10.1089/ten.tec.2018.0324 PMC6389771 30648479

[17] B. M. Sicari, V. Agrawal, B. F. Siu, C. J. Medberry, C. L. Dearth, N. J. Turner, S. F. Badylak, Tissue Eng., Part A 2012, 18, 1941. 22906411 10.1089/ten.tea.2012.0475 PMC3463275

[18] T. A. Lew, J. A. Walker, J. C. Wenke, L. H. Blackbourne, R. G. Hale, J. Oral. Maxillofac. Surg. 2010, 68, 3. 20006147 10.1016/j.joms.2009.06.006

[19] R. Gassner, T. Tuli, O. Hachl, A. Rudisch, H. Ulmer, J. Craniomaxillofac. Surg. 2003, 31, 51. 12553928 10.1016/s1010-5182(02)00168-3

[20] A. De Sousa, Br. J. Oral Maxillofac. Surg. 2008, 46, 661. 18771826 10.1016/j.bjoms.2008.07.192

[21] M. T. Conconi, P. De Coppi, S. Bellini, G. Zara, M. Sabatti, M. Marzaro, G. F. Zanon, P. G. Gamba, P. P. Parnigotto, G. G. Nussdorfer, Biomaterials 2005, 26, 2567. 15585259 10.1016/j.biomaterials.2004.07.035

[22] P. De Coppi, S. Bellini, M. T. Conconi, M. Sabatti, E. Simonato, P. G. Gamba, G. G. Nussdorfer, P. P. Parnigotto, Tissue Eng. 2006, 12, 1929. 16889522 10.1089/ten.2006.12.1929

[23] C. L. Dearth, P. F. Slivka, S. A. Stewart, T. J. Keane, J. K. Tay, R. Londono, Q. Goh, F. X. Pizza, S. F. Badylak, Acta Biomater. 2016, 31, 50. 26612417 10.1016/j.actbio.2015.11.043 PMC4728713

[24] X. K. Chen, T. J. Walters, J. Plast. Reconstr. Aesthet. Surg. 2013, 66, 1750. 24007646 10.1016/j.bjps.2013.07.037

[25] B. L. Rodriguez, E. E. Vega‐Soto, C. S. Kennedy, M. H. Nguyen, P. S. Cederna, L. M. Larkin, PLoS One 2020, 15, e0239152. 32956427 10.1371/journal.pone.0239152 PMC7505427

[26] D. S. Freedman, J. B. Schroeder, G. I. Telian, Z. Zhang, S. Sunil, J. T. Ritt, J. Neural Eng. 2016, 13, 066013. 27762238 10.1088/1741-2560/13/6/066013 PMC5796755

[27] K. Kompotis, J. Hubbard, Y. Emmenegger, A. Perrault, M. Mühlethaler, S. Schwartz, L. Bayer, P. Franken, Curr. Biol. 2019, 29, 392. 30686738 10.1016/j.cub.2018.12.007

[28] B. M. Sicari, J. P. Rubin, C. L. Dearth, M. T. Wolf, F. Ambrosio, M. Boninger, N. J. Turner, D. J. Weber, T. W. Simpson, A. Wyse, E. H. Brown, J. L. Dziki, L. E. Fisher, S. Brown, S. F. Badylak, Sci. Transl. Med. 2014, 6, 234ra258. 10.1126/scitranslmed.3008085 PMC5942588 24786326

[29] R. Herbert, J.‐W. Jeong, W.‐H. Yeo, Materials 2020, 13, 517. 10.3390/ma13030517 PMC7040651 31978977

[30] H. R. Lim, H. S. Kim, R. Qazi, Y. T. Kwon, J. W. Jeong, W. H. Yeo, Adv. Mater. 2020, 32, 1901924. 10.1002/adma.201901924 31282063

[31] Y. Liu, M. Pharr, G. A. Salvatore, ACS Nano 2017, 11, 9614. 28901746 10.1021/acsnano.7b04898

[32] H. Kim, Y. S. Kim, M. Mahmood, S. Kwon, N. Zavanelli, H. S. Kim, Y. S. Rim, F. Epps, W. H. Yeo, Adv. Sci. 2020, 7, 2000810. 10.1002/advs.202000810 PMC7404159 32775164

[33] S. Kwon, Y.‐T. Kwon, Y.‐S. Kim, H.‐R. Lim, M. Mahmood, W.‐H. Yeo, Biosens. Bioelectron. 2020, 151, 111981. 31999588 10.1016/j.bios.2019.111981

[34] B. M. Sicari, J. P. Rubin, C. L. Dearth, M. T. Wolf, F. Ambrosio, M. Boninger, N. J. Turner, D. J. Weber, T. W. Simpson, A. Wyse, Sci. Transl. Med. 2014, 6, 234ra258. 10.1126/scitranslmed.3008085 PMC5942588 24786326

[35] Z. Ahmed, J. Neural Eng. 2017, 14, 056002. 28776505 10.1088/1741-2552/aa76f2

[36] D. P. Burns, K. H. Murphy, E. F. Lucking, K. D. O'Halloran, J. Physiol. 2019, 597, 831. 30570134 10.1113/JP277443 PMC6355633

[37] A. Silvani, R. Ferri, V. Lo Martire, S. Bastianini, C. Berteotti, A. Salvadè, G. Plazzi, M. Zucconi, L. Ferini‐Strambi, C. L. Bassetti, Sleep 2017, 40, zsx029. 10.1093/sleep/zsx029 28329117

[38] M. Hadzipasic, W. Ni, M. Nagy, N. Steenrod, M. J. McGinley, A. Kaushal, E. Thomas, D. A. McCormick, A. L. Horwich, Proc. Natl. Acad. Sci. USA 2016, 113, E7600. 27821773 10.1073/pnas.1616832113 PMC5127366

[39] C.‐W. Wu, G. W. Randolph, I.‐C. Lu, P.‐Y. Chang, Y.‐T. Chen, P.‐C. Hun, Y.‐C. Lin, G. Dionigi, F.‐Y. Chiang, Gland Surg. 2016, 5, 473. 27867861 10.21037/gs.2016.09.06 PMC5106378

[40] D. McShan, P. C. Ray, H. Yu, J. Food Drug Anal. 2014, 22, 116. 24673909 10.1016/j.jfda.2014.01.010 PMC4281024

[41] S. Choi, S. I. Han, D. Jung, H. J. Hwang, C. Lim, S. Bae, O. K. Park, C. M. Tschabrunn, M. Lee, S. Y. Bae, Nat. Nanotechnol. 2018, 13, 1048. 30104619 10.1038/s41565-018-0226-8

[42] P. G. Cox, N. Jeffery, Anat. Rec. 2011, 294, 915. 10.1002/ar.21381 21538924

[43] A. W. Joe, L. Yi, A. Natarajan, F. L. Grand, L. So, J. Wang, M. A. Rudnicki, F. M. Rossi, Nat. Cell. Biol. 2010, 12, 153. 20081841 10.1038/ncb2015 PMC4580288

[44] A. Uezumi, S. Fukada, N. Yamamoto, M. Ikemoto‐Uezumi, M. Nakatani, M. Morita, A. Yamaguchi, H. Yamada, I. Nishino, Y. Hamada, K. Tsuchida, Cell Death Dis. 2014, 5, e1186. 24743741 10.1038/cddis.2014.161 PMC4001314

[45] B. Del Frari, T. Schoeller, G. Wechselberger, Microsurgery 2010, 30, 192. 19957312 10.1002/micr.20721

[46] G. M. Huemer, T. Bauer, G. Wechselberger, T. Schoeller, Microsurgery 2005, 25, 196. 15744721 10.1002/micr.20105

[47] B. Del Frari, T. Schoeller, G. Wechselberger, J. Plast. Surg. Hand Surg. 2012, 46, 204. 22747358 10.3109/2000656X.2011.624697

[48] T. Pearson, L. D. Shultz, D. Miller, M. King, J. Laning, W. Fodor, A. Cuthbert, L. Burzenski, B. Gott, B. Lyons, Clin. Exp. Immunol. 2008, 154, 270. 18785974 10.1111/j.1365-2249.2008.03753.x PMC2612717

[49] H. Wu, D. Kong, Z. Ruan, P.‐C. Hsu, S. Wang, Z. Yu, T. J. Carney, L. Hu, S. Fan, Y. Cui, Nat. Nanotechnol. 2013, 8, 421. 23685985 10.1038/nnano.2013.84

[50] S. Lee, D. Sasaki, D. Kim, M. Mori, T. Yokota, H. Lee, S. Park, K. Fukuda, M. Sekino, K. Matsuura, Nat. Nanotechnol. 2019, 14, 156. 30598525 10.1038/s41565-018-0331-8

[51] Y. S. Kim, M. Mahmood, Y. Lee, N. K. Kim, S. Kwon, R. Herbert, D. Kim, H. C. Cho, W. H. Yeo, Adv. Sci. 2019, 6, 1900939. 10.1002/advs.201900939 PMC6724359 31508289

[52] Y.‐T. Kwon, H. Kim, M. Mahmood, Y.‐S. Kim, C. Demolder, W.‐H. Yeo, ACS Appl. Mater. Interfaces 2020, 12, 49398. 33085453 10.1021/acsami.0c14193

[53] H. J. Choo, A. Cutler, F. Rother, M. Bader, G. K. Pavlath, Stem Cells 2016, 34, 2784. 27434733 10.1002/stem.2467 PMC5247404

[54] Y.‐T. Kwon, Y.‐S. Kim, S. Kwon, M. Mahmood, H.‐R. Lim, S.‐W. Park, S.‐O. Kang, J. J. Choi, R. Herbert, Y. C. Jang, Nat. Commun. 2020, 11, 1. 32651424 10.1038/s41467-020-17288-0 PMC7351733

[55] H. Kim, Y.‐S. Kim, M. Mahmood, S. Kwon, F. Epps, Y. S. Rim, W.‐H. Yeo, Biosens. Bioelectron. 2020, 173, 112764. 33190046 10.1016/j.bios.2020.112764 PMC8093317

[56] Y. T. Kwon, Y. Lee, G. K. Berkmen, H. R. Lim, H. A. Jinnah, W. H. Yeo, Adv. Mater. Technol. 2019, 4, 1900458. 33043125 10.1002/admt.201900458 PMC7546326

[57] Y.‐T. Kwon, J. J. Norton, A. Cutrone, H.‐R. Lim, S. Kwon, J. J. Choi, H. S. Kim, Y. C. Jang, J. R. Wolpaw, W.‐H. Yeo, Biosens. Bioelectron. 2020, 165, 112404. 32729524 10.1016/j.bios.2020.112404 PMC7484316

