# Supplementary material for: Real‐Time Functional Assay of Volumetric Muscle Loss Injured Mouse Masseter Muscles via Nanomembrane Electronics
Source: Adv Sci (Weinh). 2021 Jul 3;8(17):2101037. doi: 10.1002/advs.202101037 (PMC8425913; doi:10.1002/advs.202101037)
Supplement: Supplementary file 1 — Supporting Information [file ADVS-8-2101037-s001.pdf]

© 2021 Wiley-VCH GmbH

ADVANCED SCIENCE
Open Access

Supporting Information

for *Adv. Sci.*, DOI: 10.1002/advs.202101037

Development of craniofacial volumetric muscle loss model and real-time functional assay of injured muscles via nanomembrane electronics

Hojoong Kim, Young-Tae Kwon, Carol Zhu, Fang Wu, Shinjae Kwon, Woon-Hong Yeo, and Hyojung J. Choo**

Supporting Information

Development of craniofacial volumetric muscle loss model and real-time functional assay of injured muscles via nanomembrane electronics

Hojoong Kim, Young-Tae Kwon, Carol Zhu, Fang Wu, Shinjae Kwon, Woon-Hong Yeo, and Hyojung J. Choo**

Dr. H. Kim, S. Kwon, Prof. W.-H. Yeo
George W. Woodruff School of Mechanical Engineering, College of Engineering, Georgia Institute of Technology, Georgia Institute of Technology, Atlanta, GA 30332, USA
Center for Human-Centric Interfaces and Engineering, Institute for Electronics and Nanotechnology, Georgia Institute of Technology, Atlanta, GA 30332, USA

Dr. Y.-T. Kwon
Department for Metal Powder, Korea Institute of Materials Science, Changwon 51508, South Korea

C. Zhu, Dr. F. Wu, Prof. H.J. Choo
Department of Cell Biology, School of Medicine, Emory University, Atlanta, GA, 30322, USA

Prof. W.-H. Yeo
Wallace H. Coulter Department of Biomedical Engineering, Parker H. Petit Institute for Bioengineering and Biosciences, Institute for Materials, Neural Engineering Center, Institute for Robotics and Intelligent Machines, Georgia Institute of Technology, Atlanta, GA 30332, USA

E-mail: whyeo@gatech.edu (W.-H. Yeo) and hyojung.choo@emory.edu (H.J. Choo)

Note S1. Fabrication details of the wearable electronics system.

1.1 Graphene ink preparation

1. For the electrochemical exfoliation, 10 V was applied between the graphite (Alfa Aesar) and Pt foil in an electrolyte solution of ammonium sulfate ($(NH_4)_2SO_4$, Sigma-Aldrich).
2. As-exfoliated graphene was purified using deionized water (DI water) and further filtered under vacuum to remove the residuals.
3. The filtered wet powder of graphene was dispersed in DI water and concentrated to 15%.

1.2 Graphene electrode printing

1. Spin-coat PMMA (950 PMMA, Kayaku Advanced Materials) on a glass at 1000 RPM for 30 s, bake at 200°C for 2 min.
2. Atomize a polyimide (PI) ink (PI-2545, MicroSystems) dissolved in N-Methyl-2-Pyrrolidone (NMP, Sigma-Aldrich) in a 4:1 ratio in pneumatic atomizer of Aerosol jet printer, deposit using a 300-µm-diameter nozzle, and then cure at 250°C for 1 h.
3. Print 1% of graphene ink dissolved in the NMP with a 200-µm-diameter nozzle.
4. Dissolve the printed electrodes in acetone.
5. Peel off the printed graphene layer with a water-soluble tape (ASWT-2, Aquasol) from the PMMA/slide glass and put it on a silicone elastomer (1 mm thickness, 1:2 mixture of Ecoflex 00-30 and Gels, Smooth-On). Wash the tape with DI water.

1.3 Circuit fabrication

1. Spin-coat PDMS (4:1 base-curing-agent ratio) on a Si wafer at 4000 RPM for 30 s.
2. Spin-coat 1st PI layer (PI-2610, MicroSystems) at 2000 RPM for 60 s.
3. Soft bake at 100 °C for 5 min and hard bake at 250°C for 1 h.
4. Deposit 0.5 µm thickness of Cu by sputtering.
5. Spin-coat photoresist (PR, Microposit SC1813, MicroChem) at 3000 RPM for 30 s. Align with a photomask and expose UV light and develop with a developer.
6. Etch Cu with Cu etchant (APS-100, Transene.
7. Spin-coat 2nd PI layer (PI-2545) at 2000 RPM for 60 s, and soft bake at 100°C for 5 min. Hard bake at 240 °C for 1 h in a vacuum oven.
8. Spin-coat PR (AZ P4620, Integrated Micro Materials) at 2000 RPM for 30 sec, and soft bake at 90°C for 4 min. Photolithography exposing UV light with intensity of 15 mJ/cm^2 for 100 s. Develop with a developer (AZ-400K, Integrated Micro Materials) diluted with DI water (AZ-400K: DI water = 1:4).
9. Etch for via hole with reactive ion etcher (RIE).
10. Deposit 2 µm thickness of 2nd Cu by sputtering.
11. Spin-coat PR (AZ P4620) at 1500 RPM for 30 s, and soft bake at 90°C for 4 min. Photolithography exposing UV light with intensity of 15 mJ/cm^2 for 120 s and develop.
12. Etch exposed Cu with Cu etchant.
13. Spin-coat 3rd PI layer (PI-2610) at 3000 RPM for 60 s. Soft bake at 100°C for 5 min and hard bake at 240°C for 1 h in a vacuum oven.
14. Spin-coat PR (AZ P4620) at 900 RPM for 30 sec, and soft bakes at 90°C for 4 min. Photolithography exposing UV light and develop.
15. Etch exposed PI with RIE.
16. Peel off the microfabricated circuit with a water-soluble tape from the PDMS/Si wafer.
17. Mount microchip components with screen-print low-temperature solder paste.

A

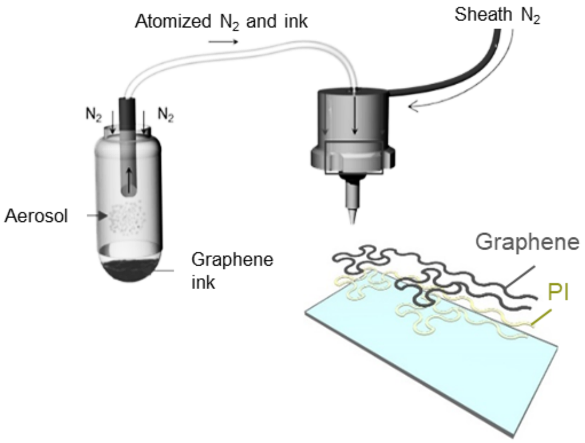

B

Factor	PI	Graphene
Solvent	N-Methyl-2-Pyrrolidone	N-Methyl-2-Pyrrolidone
Concentration	20%	1%
Viscosity (cp)	350	5
Atomization mode	Pneumatic	Ultrasonic
Atomization rate (ccm)	1000 (exhaust)<br>1100 (atomizing)	40
Sheath rate (ccm)	20	30
Nozzle diameter (μm)	300	200
Stage temperature (°C)	80	70
Sintering temperature (°C)	250	200

Figure S1. Printing process of graphene electrodes. A) Schematic illustration of the printing process of electrodes. Atomization begins in the ultrasonic vial, and a carrier gas (N_2) flows the graphene ink droplets through the tubing, the diffuser, and the deposition head, where a sheath gas focuses the particles into a narrow stream. B) Details of inks and printing parameters of PI and graphene. Resistivity and skin-contact impedance of the printed graphene were around 2×10^{-3} Ω cm and 210.5 kΩ, respectively.

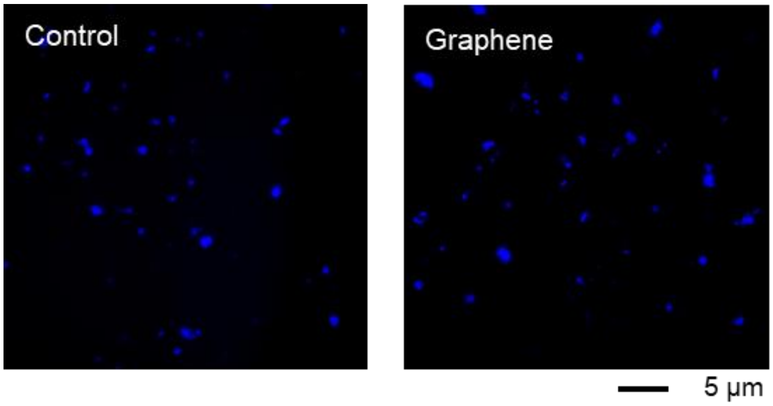

Figure S2. Biocompatibility study via cell viability measurement. Fluorescence images showing the cultured keratinocyte cells on two types of substrates, including a control (polystyrene petri dish, left) and graphene integrated on an elastomer (right). Tests used human primary keratinocyte cells cultured in an incubator at 37°C with 5% CO_2. In the incubator, the material samples were placed in a 24-well plate, and 5000 keratinocytes/cm2 were seeded. After 7 days in the incubator, keratinocyte cells were washed with phosphate-buffered saline (Fisher Chemical) and dyed with 0.1 ml of calcein blue AM (Thermo Fisher) in 0.9 ml of the cultured medium. Keratinocytes and the reagent were additionally stored in the incubator of 37°C for 10 min. The supernatant was then aliquoted in a 96-well plate for further biocompatibility.

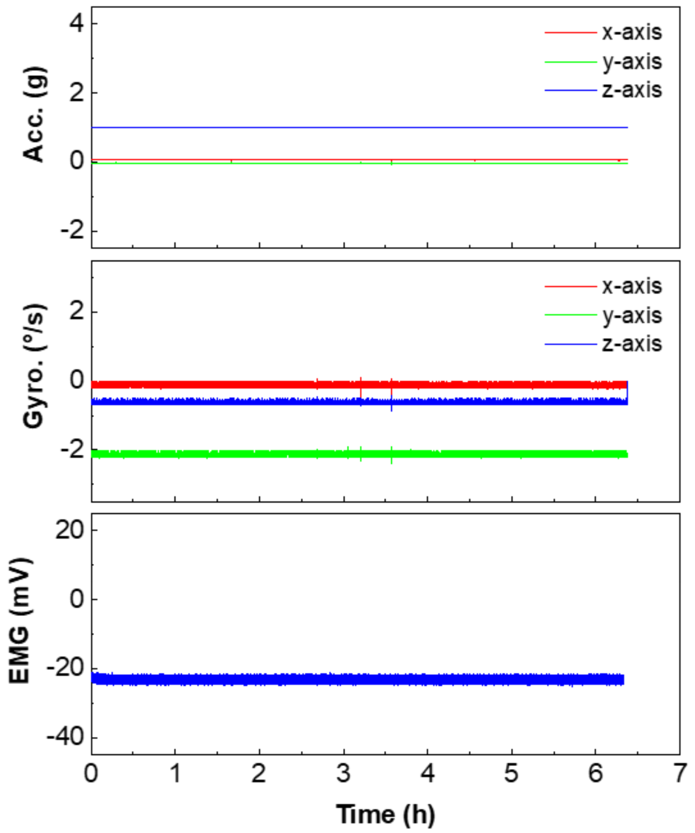

Figure S3. Maximum operation of the battery lifetime of the flexible circuit. Accelerometer (top), gyro (middle), and EMG (bottom) signals can be recorded over 6 hours using a 40 mAh lithium-ion polymer battery integrated into the circuit.

A

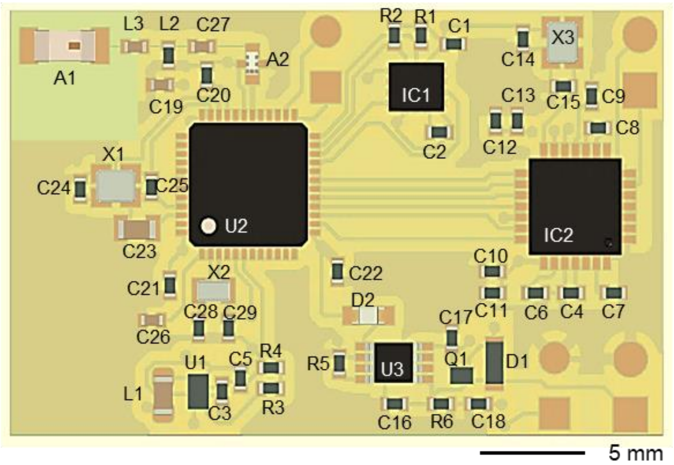

B

Cap.	Value	Cap.	Value	Cap.	Value	Res.	Value	Ind.	Value
C1	0.1 uF	C11	10 uF	C21	4.7 uF	R1	30 kΩ	L1	2.2 uH
C2	0.1 uF	C12	0.1 uF	C22	0.1 uF	R2	30 kΩ	L2	2.7 nH
C3	10 uF	C13	10 uF	C23	1 uF	R3	1 MΩ	L3	3.9 nH
C4	10 uF	C14	0.1 uF	C24	12 pF	R4	1 MΩ		
C5	10 uF	C15	15 pF	C25	12 pF	R5	2 kΩ		
C6	10 uF	C16	4.7 uF	C26	0.1 uF	R6	100 kΩ		
C7	1 uF	C17	4.7 uF	C27	1 pF				
C8	1 uF	C18	1 uF	C28	12 pF				
C9	1 nF	C19	0.1 uF	C29	12 pF				
C10	0.1 uF	C20	0.1 nF						

C

Comp.	Description
Q1	MOSFET
D1	Schottky diode
D2	LED
X1	32 MHz oscillator
X2	32 kHz oscillator
X3	32 kHz oscillator
U1	1.8 V regulator
U2	Microcontroller
U3	Charging controller
IC1	Motion sensor
IC2	Bio potential AFE
A1	2.4 GHz antenna
A2	High frequency ceramic

Figure S4. Circuit assembling information. A) The schematics of the circuit with the detailed numbering of chip components. B) Table list of RLC components (capacitors, resistors, and inductors) and C) functional parts used in the design.

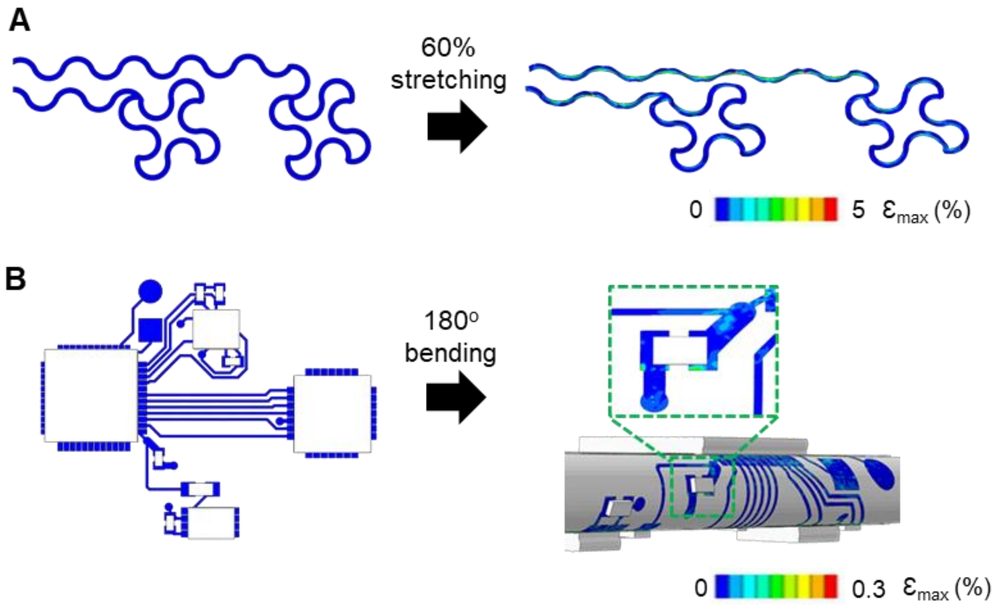

Figure S5. Computational mechanical simulation. A) Finite element analysis (FEA) results showing the mechanical compliance of printed electrodes before (left) and after 60% uniaxial stretching (right). B) FEA results of the bendable circuit upon 180°, showing no mechanical fracture.

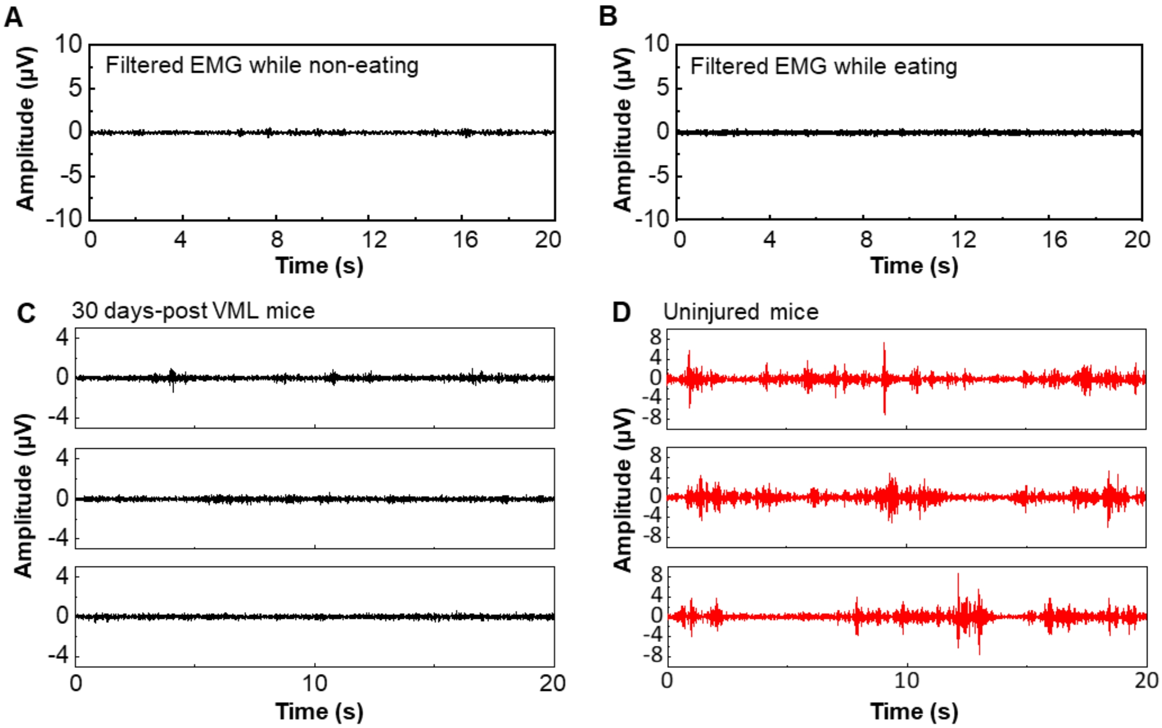

Figure S6. Filtered EMG signals of a post-VML-injured mouse. There is no signal variation for chewing behavior due to masseter muscle loss between A) non-eating and B) eating. Chewing EMG signals of 3 mice with C) post-VML-injured after 30 days and D) uninjured masseter muscle.

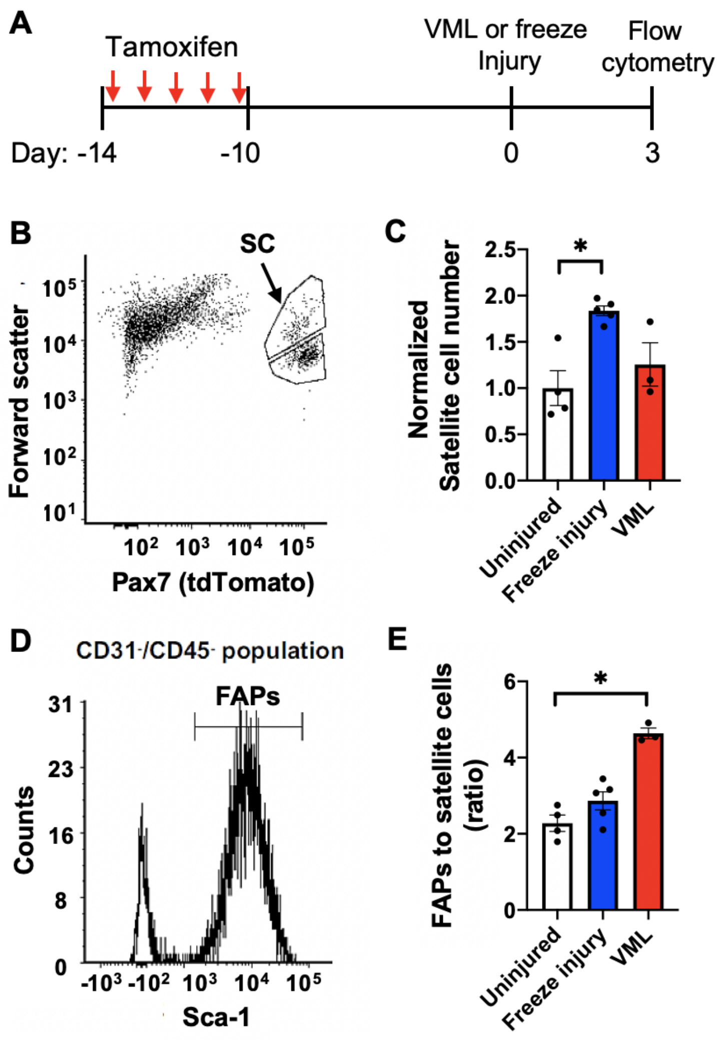

Figure S7. Dysregulated stem cells in masseter muscles at 3 days post-VML injury. (A) Scheme of experiments. Tamoxifen was injected Pax7$^{cre/ERT}$;tdTomato mice for 5 days to induce tdTomato fluorescence expression in satellite cells. VML or freeze injury was performed 10 days after tamoxifen injection. Mononucleated cells were isolated for flow cytometry analysis at 3 days after injury. (B) Representative dot plot of satellite cells, which are gated by red fluorescent protein (tdTomato), using flow cytometry. (C) Number of satellite cells from VML-injured masseter muscles is comparable with one of uninjured masseter muscles. Satellite cell numbers are normalized to average satellite cell number of uninjured muscles. Freeze-injured muscles are served as a positive control. Error bars represent standard error of the mean (SEM). (D) Representative histogram of fibroadipose cells (FAPs), which is defined by surface markers (Cd31$^-$, CD45$^-$, Sca1$^+$) using flow cytometry. (E) Ratio FAPs to satellite cells is increased in VML-injured masseter muscles. Data is analyzed 1-way ANOVA and Kruskal-Wallis method for post-hoc comparison. *$p<0.05$.

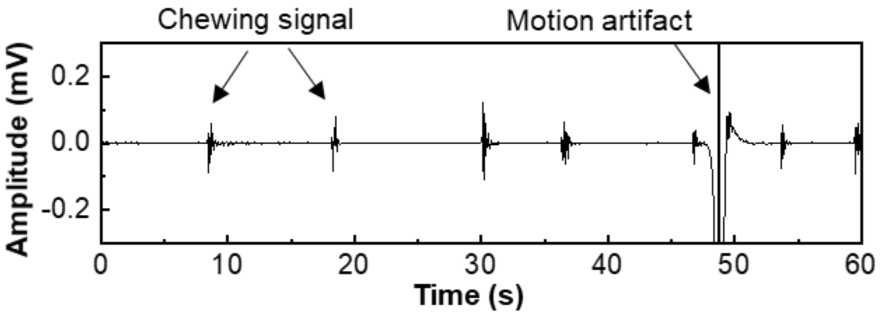

Figure S8. Filtered EMG signals of an uninjured mouse while eating. Motion artifacts make a significant and distinguishable amplitude comparing with the chewing signals.

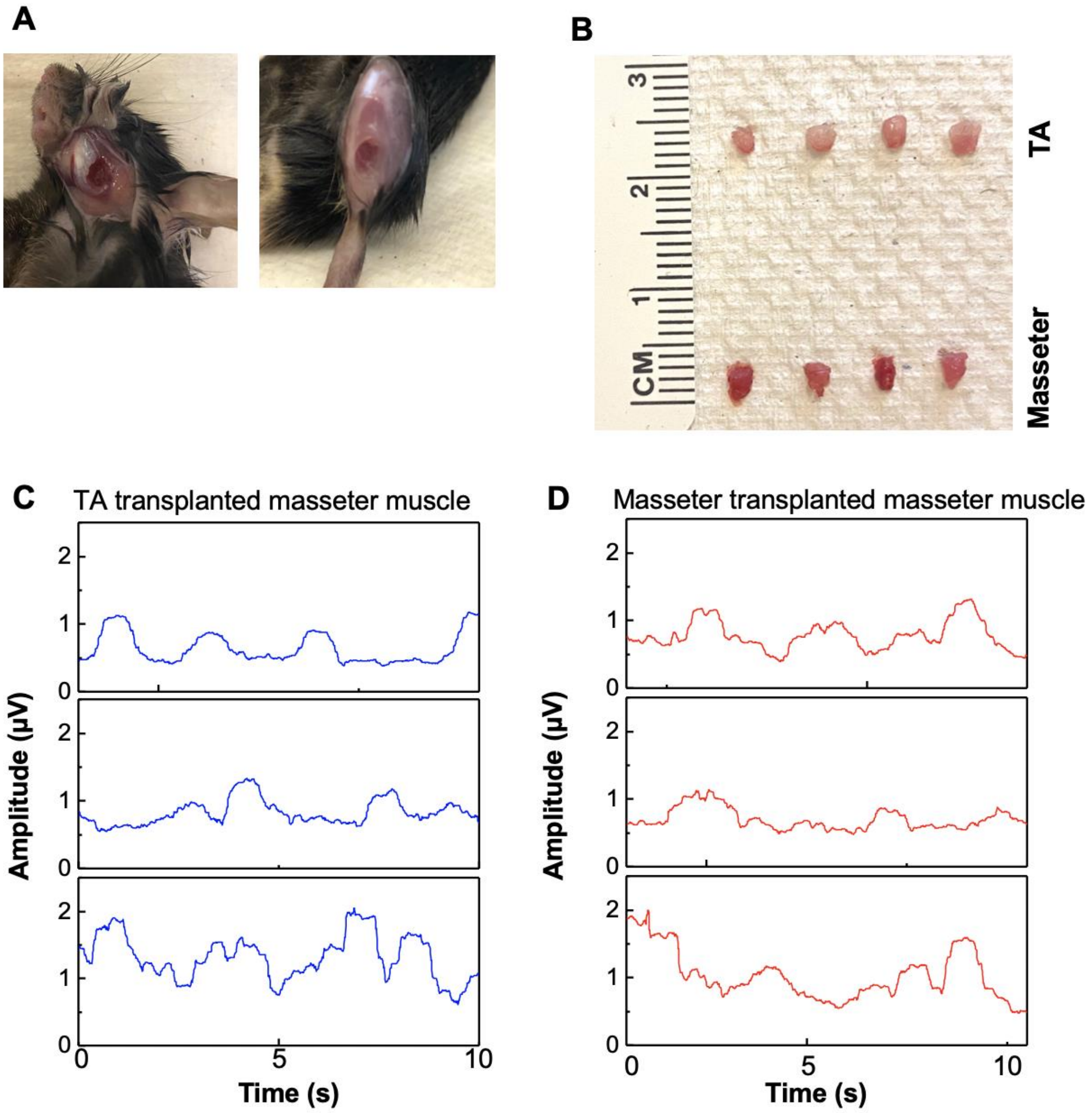

Figure S9. (A) Representative images of 3-mm punch on masseter and TA muscles of female mice. (B) Images of the biopsied masseter and TA muscles to compare general mass for transplant. Chewing RMS EMG signals of 3 mice with (C) TA muscle-transplanted and (D) masseter muscle-transplanted at craniofacial muscle area.

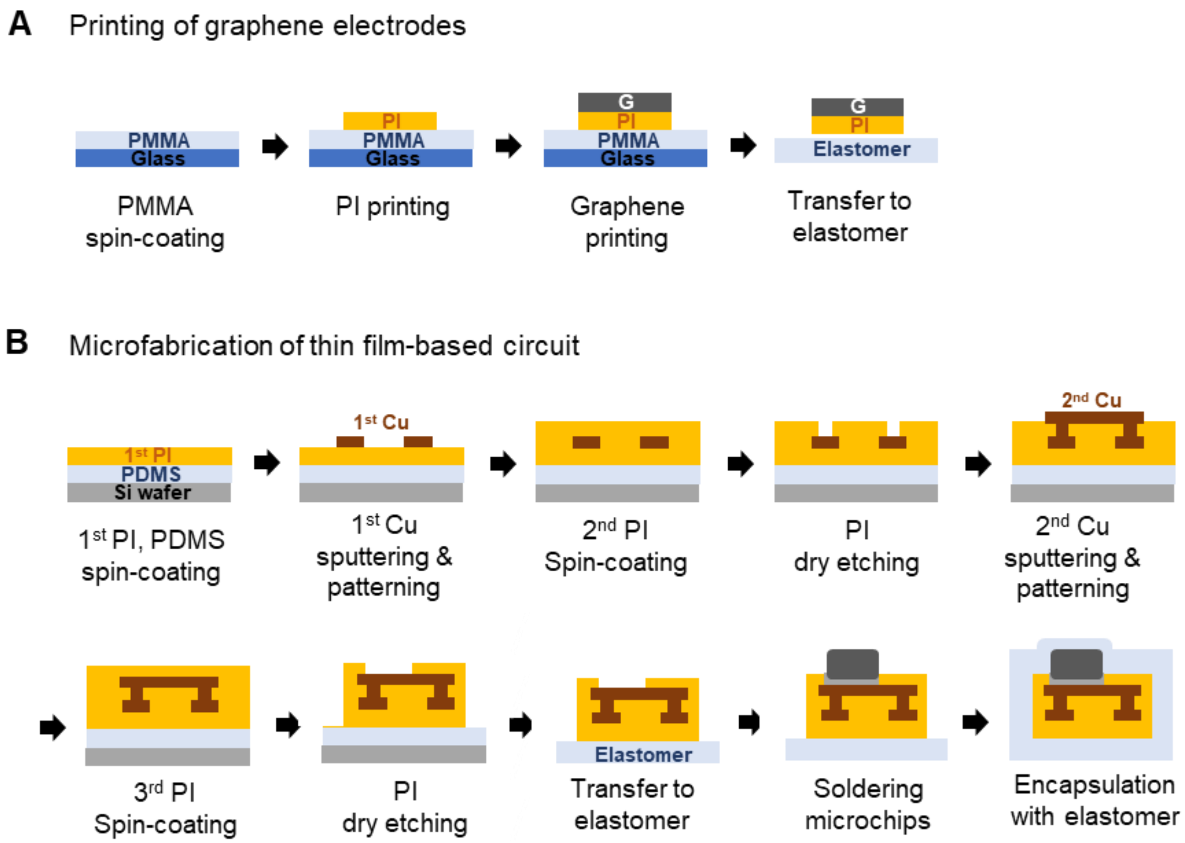

Figure S10. Schematics of the device fabrication process. A) Fabrication process of stretchable graphene membrane electrodes and B) microfabrication of thin film-based flexible circuit.

Table S1. Power analysis of animal study

Figure	P value	Effect size (d or *f)	Power	Analysis method
Figure 3B	>0.01	2.157	0.89	T-test, two independent means
Figure 3D	Not significantly different	1.527	0.44	T-test, two independent means
Figure 3E	Not significantly different	0.315	0.06	T-test, two independent means
Figure 4G	>0.01	12.523	1	T-test, two independent means
Figure 5B	Not significantly different	0.105*	0.05	T-test, two independent means
Figure 5D	>0.01	18.3*	1	F test, ANCOVA: fixed effects, main effects and interactions
Figure 5G	>0.05	7.141*	1	F test, ANCOVA: fixed effects, main effects and interactions
Figure S6C	>0.05	1.168*	0.9	F test, ANOVA: fixed effects, omnibus, one-way
Figure S6E	>0.05	3.319*	1	F test, ANOVA: fixed effects, omnibus, one-way

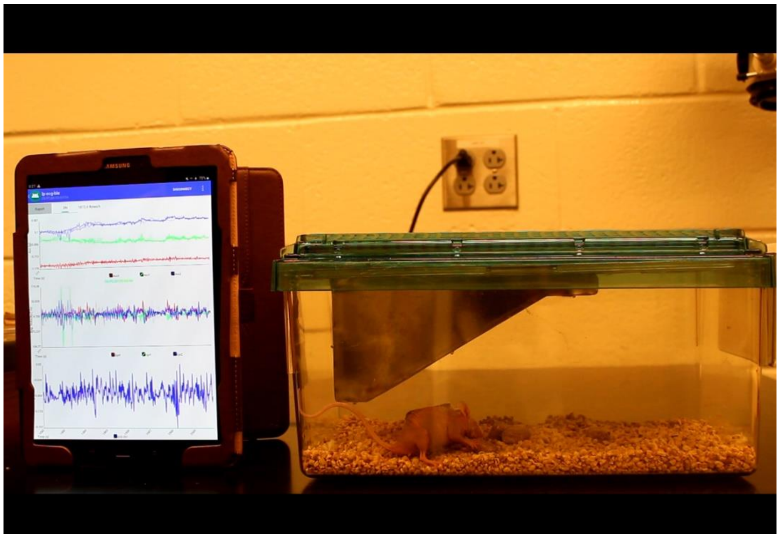

Movie S1. Wireless EMG monitoring on the masseter muscle of mouse with miniaturized portable electronics.
